# Diagnostic accuracy of post-mortem CT with targeted coronary angiography versus autopsy for coroner-requested post-mortem investigations: a prospective, masked, comparison study

**DOI:** 10.1016/S0140-6736(17)30333-1

**Published:** 2017-07-08

**Authors:** Guy N Rutty, Bruno Morgan, Claire Robinson, Vimal Raj, Mini Pakkal, Jasmin Amoroso, Theresa Visser, Sarah Saunders, Mike Biggs, Frances Hollingbury, Angus McGregor, Kevin West, Cathy Richards, Laurence Brown, Rebecca Harrison, Roger Hew

**Affiliations:** a University of Leicester, East Midlands Forensic Pathology Unit, Leicester Royal Infirmary, Leicester, UK; b Radiology Department, Leicester Royal Infirmary, Leicester, UK; c Pathology Department, Leicester Royal Infirmary, Leicester, UK; d Department of Imaging, Narayana Health City Campus, Bangalore, India; e Department of Medical Imaging, Toronto General Hospital, Toronto, ON, Canada; f Histopathology Department, Royal Devon and Exeter NHS Foundation Trust, Church Road, Exeter, Devon, UK

## Abstract

**Background:**

England and Wales have one of the highest frequencies of autopsy in the world. Implementation of post-mortem CT (PMCT), enhanced with targeted coronary angiography (PMCTA), in adults to avoid invasive autopsy would have cultural, religious, and potential economic benefits. We aimed to assess the diagnostic accuracy of PMCTA as a first-line technique in post-mortem investigations.

**Methods:**

In this single-centre (Leicester, UK), prospective, controlled study, we selected cases of natural and non-suspicious unnatural death referred to Her Majesty's (HM) Coroners. We excluded cases younger than 18 years, known to have had a transmittable disease, or who weighed more than 125 kg. Each case was assessed by PMCTA, followed by autopsy. Pathologists were masked to the PMCTA findings, unless a potential risk was shown. The primary endpoint was the accuracy of the cause of death diagnosis from PMCTA against a gold standard of autopsy findings, modified by PMCTA findings only if additional substantially incontrovertible findings were identified.

**Findings:**

Between Jan 20, 2010, and Sept 13, 2012, we selected 241 cases, for which PMCTA was successful in 204 (85%). Seven cases were excluded from the analysis because of procedural unmasking or no autopsy data, as were 24 cases with a clear diagnosis of traumatic death before investigation; 210 cases were included. In 40 (19%) cases, predictable toxicology or histology testing accessible by PMCT informed the result. PMCTA provided a cause of death in 193 (92%) cases. A major discrepancy with the gold standard was noted in 12 (6%) cases identified by PMCTA, and in nine (5%) cases identified by autopsy (because of specific findings on PMCTA). The frequency of autopsy and PMCTA discrepancies were not significantly different (p=0·65 for major discrepancies and p=0·21 for minor discrepancies). Cause of death given by PMCTA did not overlook clinically significant trauma, occupational lung disease, or reportable disease, and did not significantly affect the overall population data for cause of death (p≥0·31). PMCTA was better at identifying trauma and haemorrhage (p=0·008), whereas autopsy was better at identifying pulmonary thromboembolism (p=0·004).

**Interpretation:**

For most sudden natural adult deaths investigated by HM Coroners, PMCTA could be used to avoid invasive autopsy. The gold standard of post-mortem investigations should include both PMCT and invasive autopsy.

**Funding:**

National Institute for Health Research.

## Introduction

Studies^1^, ^2^, ^3^, ^4^, ^5^ of post-mortem investigations of both natural and unnatural death have shown that the diagnostic yield increases when post-mortem CT (PMCT)^1^ is added to autopsy, specifically for detection of fractures, haemorrhage, and gas collections, such as pneumothorax.^2^, ^3^, ^4^ The use of PMCT as an alternative to autopsy was first suggested in 1994,^5^ but it has not yet been established.^6^, ^7^, ^8^, ^9^, ^10^

**Figure 1:**
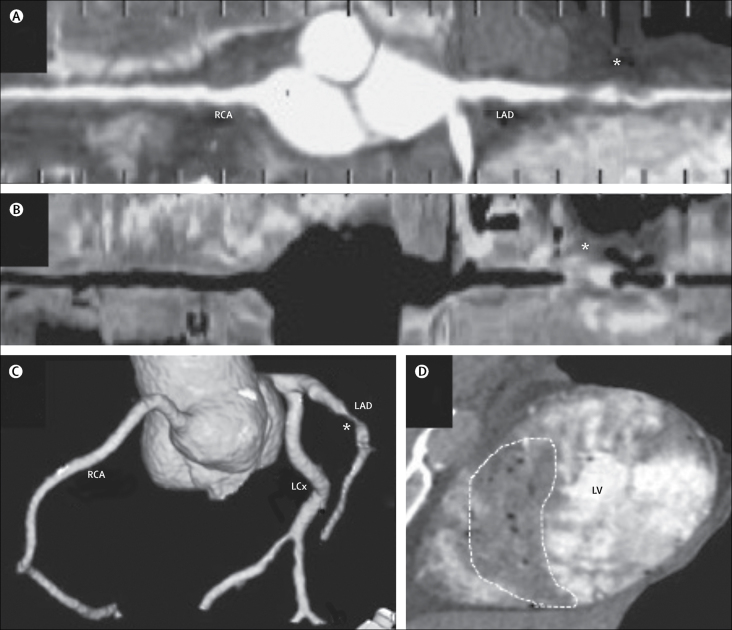
Images from post-mortem CT with targeted coronary angiography in a case of myocardial infarction Post-mortem CT with targeted coronary angiography (PMCTA) of a male ex-smoker aged 62 years with borderline type 2 diabetes who died suddenly and unexpectedly. PMCTA reconstructed images with straightened curved multiplane reconstructions of the positive (A) and air (B) contrast runs, and a 3D-volume reformat (C), all showing a critical stenosis of the proximal left anterior descending artery, as indicated by an asterisk. The myocardium showed an anteroseptal perfusion deficit, indicated by a dashed line (D). In the absence of artery calcification, these findings were diagnosed as a soft plaque occlusion leading to acute myocardial infarction. RCA=right coronary artery. LAD=left anterior descending. LCx=left circumflex. LV=left ventricle.

In 2014, 89 875 autopsies requested by Her Majesty's (HM) Coroners were done in England and Wales.^11^ Autopsy—ie, internal post-mortem examination—is the established gold standard for the investigation of cause of death; however, because of religious, cultural, economic, and workforce reasons, replacement of the invasive component of these investigations by PMCT might be preferable in some circumstances. One major problem associated with the use of PMCT is the inability to accurately diagnose coronary artery disease, the most common cause of sudden death in adults.^4^, ^6^ This problem has been addressed by the addition of angiography (PMCTA), either targeting the coronary arteries only or the whole body (figure 1).^12^, ^13^ Small, controlled studies of PMCTA have produced encouraging results, reporting good correlation, statistically and visually, with autopsy for identification of coronary artery disease.^14^, ^15^, ^16^ A feasibility study,^17^ done between 2011 and 2012, suggests this technique can reduce the frequency of autopsies; 120 cases were assessed by PMCT (of which 35 were assessed by targeted coronary PMCTA) and only 55 cases had an autopsy control. PMCT and PMCTA services are now available in a few sites across the UK, but no large, autopsy-controlled study has investigated the effectiveness of targeted coronary PMCTA as an alternative to autopsy in the investigation of sudden death in adults by HM Coroners. Other specific weaknesses of PMCT, not addressed by targeted coronary angiography, are the inability to identify pulmonary infections and emboli, non-ischaemic heart disease, gastrointestinal haemorrhage, and sepsis.^17^, ^18^

**Research in context**

**Evidence before this study**

Post-mortem CT (PMCT) has been used as an adjunct to assist in post-mortem investigations since 1983, but has also been proposed as an alternative to invasive autopsy since 1994. We searched PubMed databases (between July 3, 2009, and Nov 20, 2015) with the terms “post-mortem imaging”, “minimally (and non) invasive autopsy”, “computed tomography (CT)”, “magnetic resonance imaging (MRI)”, and “angiography and contrast media”, with no language restrictions. Discussions at the UK National Department of Health representative groups and subsequently at the International Society of Forensic Radiology and Imaging (ISFRI) revealed that several centres were doing routine PMCT as an adjunct to autopsy at the time of study initiation. Published studies mainly focused on unnatural death, but often included cases of natural death, and there was one large ongoing study of 200 cases in England and Wales proposing non-contrast-enhanced PMCT as an alternative to autopsy in natural death. These studies suggested that although PMCT is a useful adjunct to autopsy by diagnosing more pathology and particularly trauma, it has a major weakness in diagnosing parenchymal disease, and particularly in diagnosing coronary vascular disease, the most common cause of adult sudden death. Over the past decade, several teams worldwide have addressed this independently by adding angiography (PMCTA) with a variety of techniques including multiphase whole-body PMCTA, and, in the case of this study, targeted coronary angiography. These techniques have shown promise for the diagnosis of cardiovascular disease, and one English study of 120 cases showed that this approach can be used in the setting of Her Majesty's (HM) Coroners. However, this study used angiography in only about 30% of cases and autopsy control in fewer than half of cases, which was dependent on the imaging result. Currently, all studies have either involved fewer than 20 cases, were not independently autopsy-controlled, or were not applicable to the HM Coroner system in England and Wales. Therefore, to our knowledge, no large, fully autopsy-controlled study investigating PMCTA as an alternative to autopsy in the investigation of adult sudden death has been published.

**Added value of this study**

Assessment of both autopsy and PMCT errors was possible because they were considered independently, both with access to all information that was available non-invasively, which allowed an independent gold standard to be created on the basis of the positive results from both tests. We showed that PMCTA can provide a cause of natural death in 92% of cases investigated by the HM Coroner, without missing trauma, occupational lung disease, or reportable disease, with no significant difference in the overall population data for cause of death. The frequency of discrepancies with the gold standard (errors) between the two approaches was not significantly different, but the types of discrepancy were different: PMCTA was better at identifying trauma and haemorrhage, and autopsy better at identifying pulmonary thromboembolism.

**Implications of all the available evidence**

For most natural and some trauma-related adult deaths investigated by HM Coroners in England and Wales, in which the degree of evidence required is on the balance of probabilities, PMCTA can be used to avoid invasive autopsy. Furthermore, when a higher burden of proof is required, the gold standard of post-mortem investigation should include both PMCTA and invasive autopsy.

We investigated the effect of replacing autopsy with targeted coronary PMCTA (referred to as PMCTA^1^ for the rest of our Article). Our aims were to assess the diagnostic accuracy of cause of death derived by PMCTA, the sensitivity and specificity of PMCTA in identifying common causes of death, the potential reduction in the frequency of autopsies for HM Coroner cases, and the potential effect of introducing PMCTA on statistics for overall cause of death.

## Methods

### Study design and cases

This prospective, controlled comparison study was done at a single centre in Leicester, UK. The appendix (p 3) shows our study flow chart. The study protocol was approved by the local research ethics committee (04_Q2501_64).

We prospectively selected study cases, including both natural and non-suspicious unnatural deaths, referred and authorised for invasive autopsy examination by HM Coroners from two jurisdictions (North and South Leicestershire, covering the whole region). The cases were selected in a consecutive manner on the basis of the first suitable referral for a post-mortem investigation received by a secure fax machine on a chosen study day. Study days were chosen on the basis of staff availability and other ongoing trials. Cases were excluded if they were aged younger than 18 years, known to have had a transmittable disease (eg, tuberculosis, HIV, or hepatitis C), or weighed more than 125 kg (because of CT scanner weight limit). Cases were recruited only if oral consent by telephone was gained for study participation from the relatives of the deceased. ^19^ Time of death was recorded if known. If a case met any of the exclusion criteria, or if consent could not be obtained, then either the next suitable referral was chosen or no case was recruited on that particular study day.

### Procedures

All cases were given a unique anonymisation code at study entry. This code was used as the case identifier for all image data and other relevant information that related to the post-mortem examination, including the autopsy report and any further tests done by the study coordinator.

Our detailed protocol is shown in the appendix (p 2). All study cases were cannulated within a mortuary licensed by the Human Tissue Authority, principally with a 14Fr silicone-coated, male urinary catheter (Bardia Foley catheter, Bard Medical, Crawley, UK) inserted into the ascending aorta, just above the aortic valve, via the left common carotid artery, by means of a cutdown procedure.

PMCT and PMCTA were done with a Toshiba Aquilion 64 slice scanner (Toshiba Medical Systems, Otawara, Japan) by radiographers experienced in forensic imaging (led by CRo). Pre-contrast scans were done in three overlapping blocks: head and neck; chest, abdomen, and pelvis; and pelvis and legs. Contrast runs through the heart used five separate sequences, with air for the first three sequences, followed by two sequences of Urografin 150 mg/mL (positive contrast; Bayer, Newbury, UK) diluted 1/10. The first 150 cases involved manual injection via a standard 60 mL bladder syringe with gentle constant hand pressure and the remaining cases involved use of a Medrad Stellant dual head pump injector system (Medrad UK Ltd, Ely, UK), with the intention to improve coronary artery filling and distension.

Invasive autopsy was planned for the next working day after PMCTA and any delay was recorded. The pathologists were not aware of the PMCTA findings, unless PMCT showed potential risk to them, such as tuberculosis infection. All pathologists (MB, FH, AM, KW, CRi, LB, RHa, and RHe) agreed to take part in the study and undertake the autopsy following standard practice (Royal College of Pathologists generic and cause of death specific guidelines).^20^ The history of the case compiled by the HM Coroner was available to the pathologist. Any further investigation—eg, supplementary laboratory investigations, including toxicology and histology—required explicit permission from the HM Coroner. The decision about the number and extent of any further tests was made during the investigation, at the discretion of the pathologist on duty.

All PMCT and PMCTA images were reported initially by a radiologist (BM) who had 4 years' experience in PMCT at the beginning of the study (involving more than 200 PMCT cases in which the findings could be compared with those from the autopsy). For most cases, PMCT was also reported by a cardiac radiologist (VR and MP) and a consensus report generated. This initial PMCTA report also made use of the coroner-compiled case history provided to the pathologist undertaking the autopsy.

All cases were re-reported by the radiologist together with a pathologist (GNR) who had experience of using PMCT but who was not involved in the autopsy, with any additional external examination and clinical history information extracted from the autopsy report. If possible, in each case a cause of death was recorded by the radiologist and pathologist in consensus on the basis of a balance of probabilities, which is the principle of English and Welsh HM Coronial Law. The PMCTA reviewers also made a decision termed triage to autopsy if they had low confidence in the PMCTA-derived cause of death. After this stage, the internal examination findings and autopsy-identified cause of death were revealed to the PMCTA reviewers.

Two anonymous reports were created by the study manager (TV): a full report and a reduced PMCTA report that excluded the internal examination findings, but included external examination details and any additional medical history. Toxicology and biochemistry information was given to the radiologist if requested and available. Likewise, histology information was given to the radiologist for the PMCTA report after direct request, and only for tissue that could easily be accessed by CT-guided biopsy. The full report with autopsy-derived cause of death was used for comparison (by BM, GNR, and CRo) with the equivalent PMCTA-derived report.

We took the autopsy-identified cause of death and findings to be the gold standard unless modified by one of four factors (appendix p 4): (1) PMCTA showed a clear and incontrovertible finding, such as fracture or major haemorrhage, for which PMCT can be considered specific; (2) a clinically significant finding was identified by PMCT, increasing the relevance of similar findings on autopsy, adding to the cause of death (eg, autopsy detects a finding, but PMCT shows it to be more extensive than appreciated at autopsy); (3) specific pre-mortem investigations and findings that were not acknowledged in the autopsy report that contradict the autopsy diagnosis; or (4) cause of death was constructed incorrectly on the basis of the autopsy findings.^21^ We assessed autopsy errors resulting from one or more of these factors in the discrepancy analysis.

When a discrepancy existed between the findings of the two investigations, the PMCT images, the autopsy report, and any previously available ante-mortem laboratory and imaging investigations were reviewed (by GNR, BM, and CRo) to establish the nature of the discrepancy and the validity of the autopsy findings. Differences were categorised into major (eg, overlooked clinically significant trauma or any potentially fatal finding), minor (eg, same findings, but different cause of death given), no discrepancy (eg, same causes of death, but given in a different order), or were recorded separately and not included in the discrepancy analysis (appendix pp 4–8). A trivial change is when the gold standard result is changed from the autopsy result, but does not result in a discrepancy between the autopsy and gold standard.

### Outcomes

The primary endpoint of this study was to assess, in cases in which PMCTA could give a cause of death, the accuracy of this cause of death diagnosis against a gold standard of autopsy findings, modified by PMCTA findings only if additional substantially incontrovertible findings were identified. Secondary endpoints included the success of coronary angiography (defined by successful catheter placement with subsequent opacification of coronary arteries by contrast), the proportion of cases in which cause of death could be given by PMCTA, the sensitivity and specificity of PMCTA and autopsy for specific common diseases, including unexpected trauma, ischaemic heart disease, and pulmonary thromboembolism, and the potential effect of PMCTA on overall mortality statistics.

### Statistical analysis

All cases were included in the assessment of technical success of the procedure. Cases in which the cause of death was known and clearly traumatic—eg, road traffic collisions, hanging, and gunshot wounds—were excluded from the analysis of the diagnostic accuracy of PMCTA in determining the cause of death. Demographic data are presented as mean (SD), median (IQR), and range. We used McNemar's test to calculate p values for paired proportions and χ^2^ tests for p values of unpaired proportions using SPSS version 22.0. We calculated the sensitivity and specificity of both autopsy and the PMCTA-first approach (ie, PMCTA findings, or autopsy results if PMCTA did not give a cause of death) against the gold standard. A diagnosis was only considered to be a false positive if the gold-standard investigation neither gave it as a cause of death nor mentioned it in the findings. For example, if PMCTA gave a diagnosis of cardiac disease in a patient who died of pulmonary thromboembolism but who also had clinically significant cardiac disease in the autopsy findings, the cardiac diagnosis for cause of death on PMCTA counted as a false negative for pulmonary thromboembolism but not a false positive for cardiac disease. We calculated sensitivity and specificity of PMCTA and autopsy for four diagnoses (pulmonary thromboembolism, trauma, respiratory disease, and cardiac disease), and provide the accompanying 95% CIs (MedCalc version 15.0).

### Role of the funding source

The funder of the study had no role in the study design, data collection, data analysis, data interpretation, or writing of the report. The co-chief investigators (GNR and BM) had full access to all the data in the study and had final responsibility for the decision to submit for publication.

## Results

Between Jan 20, 2010, and Sept 13, 2012, we selected 241 cases for the study. No exclusions occurred as a result of weight or infectious disease. Details of the few cases for which consent was not obtained before selection have been published separately.^19^ Cases were aged 18–96 years; 158 (66%) were men and 83 (34%) women (table 1). PMCT was successful in all 241 cases. PMCTA was successful in 204 (85%) cases, with success increasing to 127 (90%) of 141 cases if the first 100 cases (which we consider as early experience) were excluded. No adverse events occurred to the scanned bodies.

**Table 1:** Demographic characteristics of study populations and success and features of PMCTA and autopsy

		**All cases (n=241)**	Diagnostic accuracy population (n=210)*
Age, years			
Median (range)		69 (18–96)	72 (26–96)
Mean (SD)		66 (19)	69 (16)
Sex			
	Men	158 (66%)	132 (63%)
	Women	83 (34%)	78 (37%)
PMCTA success			
	Fail†	37 (15%)	29 (14%)
	Successful	204 (85%)‡	181 (86%)‡
	Poor	14 (6%)	12 (6%)
	Good	190 (79%)	169 (80%)
Death-to-scan interval, h			
	Known§	NA	169
	Mean (SD)	NA	45 (27)
	Median (range)	NA	37 (8–144)
Day autopsy was done¶			
	Day 1	NA	1 (<1%)
	Day 2	NA	193 (92%)
	Day 4	NA	13 (6%)
	Day 6	NA	3 (1%)
Reporting of initial PMCTA report			
	Single	NA	51 (24%)
	Consensus	NA	159 (76%)

Data are n (%), unless otherwise specified. PMCTA=post-mortem CT with targeted coronary angiography. NA=not analysed.

* Excluding cases with clear traumatic cause of death (n=24), with no autopsy report at the time of analysis (n=4), or for which the autopsy was undertaken by the trial team (n=3).

† Failures of PMCTA (37 cases) were due to catheter progression into the descending aorta in 20 (54%) cases, difficult vascular anatomy or disease in ten (27%) cases, catheter failure in three (8%) cases, a different protocol in three (8%) cases, and abandoned procedure as a result of suspected tuberculosis in one (3%) case. Poor angiography was due to poor balloon seal in eight early cases, which was corrected by use of a bigger balloon, two cases of failure to follow correct contrast injection protocol, one case of failed image archive, one case of coronary artery bypass grafting, one case of catheter failure, and one case of catheter progression into the left ventricle

‡ Success improves to 90% if first 100 cases are excluded

§ Time of death known within 24 h

¶ PMCTA was done on day 1.

24 cases referred with a clear traumatic cause of death were excluded from the accuracy analysis (15 road-traffic and overt trauma deaths, eight deaths caused by hanging, and one self-inflicted gunshot wound; table 1) because, as expected, no difference occurred between the autopsy-diagnosed and PMCTA-diagnosed causes of death in these cases. A further seven cases were excluded from this analysis, either because no autopsy report was available at the time of analysis (four cases) or they were unmasked (three cases in which autopsy was done by the same team who reported the PMCTA). Therefore, the study group included in the diagnostic accuracy analysis consisted of 210 cases.

In six (3%) cases, the PMCTA reporting team received inappropriate information as part of the extended clinical and external examination details received. In one of these cases, toxicology details were supplied to the PMCTA reporting team, despite not being requested. However, the toxicology information was normal and did not contribute to findings. In five (2%) cases, histology information was also made available to the PMCTA reporting team when not requested. We did not consider these errors to have had an effect on the cause-of-death decision in these cases. Two of the cases already had a pre-mortem diagnosis of asbestosis and mesothelioma, and in the three other cases, the unrequested histology information had no effect on the initial cause-of-death decision (two were cardiac related and for one the histology suggested pulmonary infection, which was ignored by the radiologist, who gave a single diagnosis of end-stage fibrotic lung disease rather than infection).

Full results for histology and toxicology testing are in the appendix (pp 9–11). Regardless of whether done or not, for 42 (20%) cases the PMCTA reporting team requested either toxicology or biochemistry results on the basis of the case's history, or histology or microbiology results (if they could be obtained by CT-guided biopsy) on the basis of the history or PMCTA scan. The frequency of PMCTA discrepancies in these cases compared with the whole group were not significantly different (p=0·90; appendix p 11). In 40 (19%) cases, predictable toxicology or histology testing accessible by PMCT informed the result.

**Figure 2:**
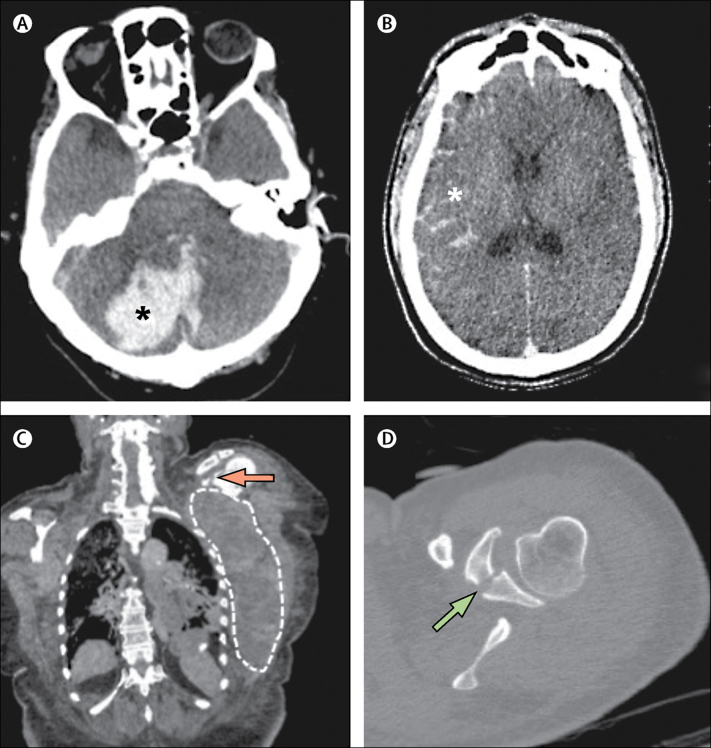
Images from post-mortem CT with targeted coronary angiography in three cases of haemorrhage (A) Axial brain image in a 73-year-old woman who collapsed and then had a cardiac arrest after a short interval. The autopsy report described a small amount of subarachnoid blood and normal cerebral cortex, but did not describe the cerebellum, whereas post-mortem CT with targeted coronary angiography (PMCTA) showed a clear clinically significant cerebellar haemorrhage, as indicated by an asterisk. Autopsy gave coronary artery disease as the cause of death, which, although also detected by PMCTA, was clearly incorrect. (B) Axial brain image of a 33-year-old man with type 1 diabetes and alcohol addiction. Toxicology showed evidence of clinically significant diabetic ketoacidosis, and both PMCTA and autopsy give the primary cause of death as diabetic ketoacidosis, but autopsy failed to report the clear subarachnoid haemorrhage, as indicated by an asterisk, which although not extensive enough to definitely cause death, might have substantially contributed to death. (C, D) An 84-year-old woman who was taking anticoagulation treatment with documented declining haemoglobin concentrations in the days leading to her death. She died from myocardial insufficiency secondary to hypovolaemia and anaemia agreed on both autopsy and PMCTA. However, autopsy did not find a bleeding source and attributed it to gastric erosions. PMCTA clearly showed a left scapula fracture (arrow) with approximately 1 L of blood in the left chest wall (dashed line).

**Figure 3:**
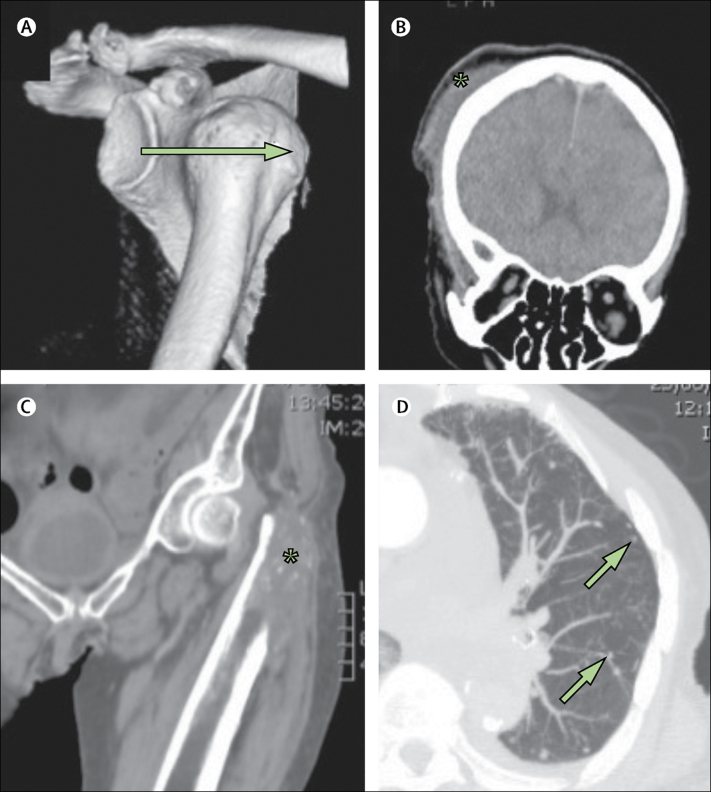
Images from post-mortem CT with targeted coronary angiography in two cases of trauma (A, B) An 86-year-old woman who was found dead in the rear doorway to her home on a cold day in February. PMCTA agreed with autopsy on the presence of ischaemic heart disease, but autopsy failed to report the trauma, which was potentially relevant in this case. (A) 3D-bone reconstruction with anterior dislocation of the shoulder (arrow shows the direction of dislocation). (B) Coronal brain multiplanar reconstruction image with subcutaneous haematoma (*). (C, D) A 91-year-old woman with an agreed primary cause of death of myocardial insufficiency due to aortic stenosis. However, PMCTA recorded in part 2 of the death certificate (associated conditions) an (C) acute pathological fracture of the left femur (asterisk) and (D) lung metastases (arrows), not reported on autopsy, which were thought likely to have acutely exacerbated her chronic cardiac condition. PMCTA=post-mortem CT with targeted coronary angiography.

The gold-standard cause-of-death diagnosis was taken from the autopsy report verbatim in 161 (77%) of 210 cases. Trivial changes to the autopsy findings, which were not deemed to affect the discrepancy analysis, were made in 23 (11%) cases. 26 (12%) non-trivial changes were made (table 2, Figure 2, Figure 3).

**Table 2:** Number of cases in which the gold standard outcome was different from the autopsy finding, and reasons for the change

		**Extent of difference**		
		Major	Minor	Trivial
Total autopsy errors		9	17	23
Clear PMCTA finding				
	Haemorrhage	2	1	..
	Trauma	4	1	..
	Trauma and haemorrhage	1	..	..
	Perforated viscous (pneumoperitoneum and fluid)	1	1	..
	Aspirated gastric contents	..	1	..
Review of autopsy report with PMCTA				
	Review of autopsy report supported by PMCTA findings	1	10	7
	PMCTA finding, and imaging and investigations before death	..	3	..
Review of autopsy report				
	Changed order of causes of death	..	..	8
	Removed factors of low clinical significance	..	..	8

PMCTA=post-mortem CT with targeted coronary angiography.

A cause of death was given by PMCT or PMCTA in 193 (92%) cases. Of these cases, the PMCTA reporting team had low confidence in 19 (10%) cases in which, despite giving a cause of death, they would still have triaged to autopsy (appendix p 12).

Table 3 shows the number of major and minor discrepancies with the gold standard cause of death for PMCTA and autopsy. PMCTA had slightly more discrepancies with the gold standard than did autopsy, but this difference was not statistically significant (p=0·65 for major discrepancies and p=0·21 for minor discrepancies). In the 193 cases for which a cause of death was given, major errors occurred in 12 (6%) cases for PMCTA and nine (5%) cases for autopsy (table 3). Exclusion of the 19 triage to autopsy cases did not significantly improve the occurrence of major errors in the PMCTA diagnosis (11 [6%] of 174; p=0·97). Therefore, we used all 193 cases in which PMCTA gave a cause of death for further comparisons.

**Table 3:** Discrepancies in cause of death against the gold standard

	**Cases for which cause of death given by PMCTA (n=193)**	**Cases for which cause of death given by PMCTA, excluding low confidence cases (n=174)**
**Major discrepancy**		
PMCTA	12 (6%)	11 (6%)
Autopsy	9 (5%)	9 (5%)
**Minor discrepancy**		
PMCTA	21 (11%)	17 (10%)
Autopsy	13 (7%)	12 (7%)

Discrepant findings were recorded per case, not per finding. Minor discrepancies in both the PMCTA and autopsy diagnosis were apparent in six cases.

PMCTA=post-mortem CT with targeted coronary angiography.

Table 4 shows a breakdown of discrepancies with the gold-standard diagnosis. This breakdown also includes cases with missed findings that were relevant to the cause of death investigation, such as an acute wrist fracture seen on PMCT in a case possibly relating to uncontrolled epilepsy, and a potentially lethal coronary artery anatomy variant seen on PMCTA (both considered minor discrepancies). PMCTA was significantly worse in the diagnosis of pulmonary thromboembolism than was autopsy, and minor discrepancies with regard to the diagnosis of respiratory disease were more common with PMCTA than with autopsy. Autopsy was significantly worse than PMCTA in the identification of trauma and haemorrhage (table 4). The sensitivity and specificity calculations for identification of pulmonary thromboembolism and trauma also found similar results (appendix p 13). Analysis of the cause of death statistics showed that PMCTA plus autopsy as required would lead to fewer cases of pulmonary thromboembolism being reported, and autopsy alone would lead to fewer cases of trauma being reported, compared with our gold standard (based on PMCTA and autopsy). However, these differences were not statistically significant (p=0·31 for trauma and p=0·68 for pulmonary thromboembolism; appendix p 14).

**Table 4:** Discrepancies with the gold standard in cause of death or clinically significant findings of the cause of death

		**Discrepancy between PMCTA and gold standard**		**Discrepancy between autopsy and gold standard**		**p value**
		Major	Minor	Major	Minor	
**Autopsy diagnosis more accurate**						
Pulmonary thromboembolism		..	..	..	..	0·004
	Sole diagnosis	1	0	0	0	..
	With comorbidity	4	1	0	0	..
	False positive	2	1	0	0	..
False positive or negative respiratory disease		2	7	0	3	..
Gastrointestinal haemorrhage with comorbidities		1	0	0	0	..
Missed cerebral infarct		1	0	0	0	..
Myocardial infarction		1	0	0	0	..
Source of sepsis not identified		0	1	0	0	..
**PMCTA diagnosis more accurate**						
Clinically significant trauma with or without haemorrhage		0	0	6	2	0·008
Missed cerebral haemorrhage		0	0	2	0	..
Wrong site of haemorrhage		0	1	0	1	..
Clinically significant coronary anatomy variant		0	0	0	2	..
**No overt difference between autopsy and PMCTA diagnosis**						
False positive or negative heart disease		0	9	0	8	..
Aspiration of gastric contents*		0	1	0	2	..
Pancreatitis or other abdominal disease*		0	2	0	0	..
Perforated viscus*		0	0	1	1	..
**Not included in discrepancy analysis**						
Pleural fluid not reported		..	1	..	30	..
Rib fractures probably related to resuscitation not reported		..	1	..	21	..
Coronary artery disease given instead of myocardial infarction as cause of death		..	16	..	..	..

PMCTA=post-mortem CT with targeted coronary angiography.

* No significant difference (separately and if all abdominal diseases considered together).

## Discussion

The results of our study show that PMCTA provided a cause of death in 92% of cases. In 11% of these cases, a major difference occurred between the autopsy or PMCTA findings and the gold standard. However, in nearly half of these cases (5% of all cases) PMCTA showed clear, clinically significant findings not reported by autopsy, which led to a change in our gold-standard diagnosis. The presumed occurrence of major errors was not significantly different between the tests. However, causes of discrepancy with the gold-standard method were different between PMCTA and autopsy. PMCT was better at detecting trauma and clinically significant soft-tissue haemorrhage, and autopsy was better at detecting pulmonary thromboembolism and respiratory disease. This outcome is consistent with previous reports,^22^, ^23^ and supports our belief that the new gold standard for post-mortem investigation is PMCT (with targeted coronary angiography) followed by autopsy.

Our study is unique in that the PMCTA and autopsy findings are reported separately, allowing a gold standard to be created independently; the study was set up prospectively with permission from HM Coroners and consent from the next of kin to have an independent PMCT investigation. In other similar international studies,^4^, ^14^ PMCT is done before autopsy on the instruction of the investigating authority, and the PMCT results have to inform the autopsy for legal and ethical reasons.

The occurrence of minor discrepancies was also not significantly different between PMCTA and autopsy. Autopsy did have numerically fewer minor discrepancies than did PMCT, but this outcome was to be expected because the gold-standard diagnosis was, by default, taken from the autopsy findings and, for minor discrepancies, PMCTA was less likely to be considered definitive enough to change the gold standard.

We believe that one of the most important findings is that if PMCTA were used as a first-line technique in post-mortem investigation, as many as 92% of autopsies might be avoided without missing clinically significant trauma (and therefore most types of unnatural death that could be identified at autopsy), occupational lung disease, or reportable disease, without significantly changing the overall population data for cause of death. Although identification of pulmonary thromboembolism is a weakness in this study of PMCTA, in general, this only applies when pulmonary thromboembolism occurs in the presence of another clinically significant pathological abnormality, such as advanced cancer or coronary artery disease. When pulmonary thromboembolism occurs in isolation with no other pathology, PMCTA will either provide the correct diagnosis or find no cause of death, in which case the body would be referred to autopsy.

Although reporters of PMCTA were asked to give a confidence judgment in their decision of cause of death, diagnostic accuracy was not significantly higher when the low-confidence group (triaged to autopsy) were excluded from the analysis. Furthermore, in many cases in which PMCTA did not give a cause of death, autopsy seemed to give only a so-called last-resort diagnosis, such as left ventricular hypertrophy or cardiomegaly, which had also been identified by PMCTA but not considered severe enough to be a definite cause of death.

We expected PMCT to be poor at identifying sepsis without obvious abscess or origin, but in this case series, PMCTA failed to diagnose the location of sepsis in only one case. This failed diagnosis was a case of meningitis in which the PMCTA report diagnosed sepsis as the cause of death, on the basis of pre-mortem history and investigations, but requested an autopsy to identify the source. This was the only case of sepsis in this study without a clear source, such as pneumonia, abscess formation, or gastrointestinal perforation.

If toxicology or histology testing is likely to be required on the basis of the history (mainly toxicology cases in this study), then these results might be delayed, and therefore the decision of whether autopsy is required might also be delayed, which might prolong the release of the body to the next of kin. For this reason, some centres would not do PMCTA as a potential alternative to autopsy in these cases, or would have to introduce rapid overnight toxicology testing.^24^ Our data show no evidence that the combination of PMCTA with toxicology or histology, or both, when requested for specific reasons, resulted in an increased frequency of discrepancy with the gold standard. However, the histology specimens used in this study, which we propose are replaceable by CT-guided biopsy, were obtained at autopsy, and therefore, would be considerably larger than standard 16G cores from a CT-guided cutting needle.

Caution should be taken in the interpretation of discrepancies as the occurrence of error for PMCTA and autopsy. PMCTA was reported by up to three observers in consensus with the opportunity to review reports, with no time constraint. However, in general, autopsies were done on routine autopsy lists for HM Coroners, with no extra time or staffing, and were therefore affected by both time constraints and numbers of cases per day. Therefore, many of the autopsy errors would probably have been avoided if more time had been available. Furthermore, PMCT might do worse in routine practice with only one observer than in our study. Autopsy, similar to PMCT, is potentially susceptible to the well recognised diagnostic error of satisfaction of search^25^—eg, dissection and further tests might be restricted once a clear, clinically significant coronary artery pathology is found, in a case with an appropriate history.

This study uses only one technique for angiography. Other techniques, such as whole-body angiography,^12^ might possibly give different results in particular circumstances, such as abdominal disease or pulmonary thromboembolism. Scans can also be reviewed immediately to decide whether angiography is required,^17^ or even what type of angiography to use. We attempted coronary angiography in all cases and did not make protocol decisions at the time of scanning because in general, scans are done outside of standard clinical hours. Therefore, we have not assessed the added value of angiography and whether it could be avoided in some cases.

In our study, the autopsy result was taken as the gold standard, unless it was clearly contradicted by imaging findings, such as by clinically significant haemorrhage or fracture, or changed by a combination of the PMCTA report and autopsy findings. The reviewers felt that the PMCTA report was probably more accurate than autopsy in many cases, and reanalysis of the results with a PMCTA as the primary gold standard, unless contradicted by clear autopsy findings, would give different results, mainly within the minor discrepancies.

No inter-reporter or intra-reporter analysis was done, but this type of analysis was not a study question. It will become a bigger issue as the use of imaging to replace autopsy in different centres, with different levels of experience and training, becomes more common.

We did not calculate the required sample size because to show statistical equivalence of PMCTA and autopsy would require considerable numbers, a gold standard independent of both PMCTA and autopsy, and a specific definition of balance of probabilities (the level of evidence required by the HM Coroner's court).^26^ Therefore, numbers were chosen within staffing and financial constraints, in line with the previous UK validation study that did not use angiography.^6^ This study does not address the financial consequences of using PMCTA as a triage test to replace autopsy. PMCT costs are highly dependent on throughput, and autopsy service costs are variable across the country and dependent on local contractual agreements.

PMCT is now becoming established as a genuine alternative to autopsy in England and Wales, and the public has a right to request it if available.^27^ Our data show that replacing the internal (autopsy) component of a full post-mortem investigation with PMCTA could replace most HM Coroners' autopsies, without missing autopsy-identifiable unnatural causes of death and reportable diseases, and would not significantly change population statistics for cause of death. PMCTA should not be reported in isolation, but imaging findings must be interpreted on the balance of probabilities, with assessors doing a full review of the medical history and an external examination, with toxicology and histology if required.
