# Supplementary material for: Diagnostic accuracy of post-mortem CT with targeted coronary angiography versus autopsy for coroner-requested post-mortem investigations: a prospective, masked, comparison study
Source: Lancet. 2017 Jul 8;390(10090):145–54. doi: 10.1016/S0140-6736(17)30333-1 (PMC5506259; doi:10.1016/S0140-6736(17)30333-1)
Supplement: Supplementary appendix [file mmc1.pdf]

THE LANCET

Supplementary appendix

This appendix formed part of the original submission and has been peer reviewed. We post it as supplied by the authors.

Supplement to: Rutty GN, Morgan B, Robinson C, et al. Diagnostic accuracy of post-mortem CT with targeted coronary angiography versus autopsy for coroner-requested post-mortem investigations: a prospective, masked, comparison study. *Lancet* 2017; published online May 24. http://dx.doi.org/10.1016/S0140-6736(17)30333-1.

Supplementary Text, Tables and Figures to Rutty, Morgan et al. Diagnostic accuracy of post-mortem Computed Tomography with targeted Coronary Angiography (PMCTA) when used as the first-line investigation for HM Coroner post-mortem investigations: prospective, blind comparison to a gold standard study.

Contents

		Page
Suppl. Text	Preparation and cannulation for PMCTA	2
Suppl. Figure S1	Study profile	3
Suppl. Text	The gold standard	4
Suppl. Text	Defining discrepancies	4
Suppl. Figure S2	Examples of discrepancy	5
Suppl. Table S1	Discrepancies defined	6
Suppl. Figure S3	Left ventricular hypertrophy (LVH)	7
Suppl. Figure S4	Cerebral infarction	8
Suppl. Results & Table S2	Reasons for toxicology / biochemistry testing	9
Suppl. Table S3	Reasons for histology / microbiology testing	10
Suppl. Table S4	Discrepancy rates when histology or toxicology were requested and performed for specific reasons	11
Suppl. Text and Table S5	Triage to autopsy decisions & failure to give a cause of death	12
Suppl. Table S6	Sensitivity and specificity for specific disease entities	13
Suppl. Table S7	Population statistics for cause of death (CoD)	14
References		15

Supplementary Text: Preparation and cannulation for Targeted Post Mortem Computed Tomography Coronary Angiography (PMCTA) scan

In this manuscript, unless clarified, PMCTA refers to Targeted Post Mortem Computed Tomography Coronary Angiography as described here, rather than other angiographic methods, for example whole body multiphase angiography techniques, [1] or angiography performed using chest compression [2] to gain circulation.

A copy of the protocol is available online (http://www2.le.ac.uk/departments/emfpu/research/NIHRprotocol_July2016.pdf).

After consent was obtained, and prior to the PMCTA scan, all study participants were prepared and cannulated within a Human Tissue Authority licensed mortuary, using a previously described method. [3,4] A variety of catheters were used for the first 12 cases and occasionally for later cases as part of catheter development, but the standard catheter used was a 14 Fr silicone-coated male urinary catheter (Bardia Foley catheter), with a ≥30ml balloon, inserted into the ascending aorta, just above the aortic valve, via the left common carotid artery by means of a cut-down procedure.

This was performed with a mortuary block placed under the middle of the shoulders, with the head turned to face right. An incision to expose the left carotid artery was made just above the medial left clavicular head. Care was taken during the dissection to avoid engorged veins especially in the area of the clavicle, as blood loss may make arterial cannulation difficult. The mortuary block was then removed prior to catheter insertion, which helps avoid the catheter proceeding down the descending aorta. The tip of the catheter was aimed towards the right axilla. Obstruction to catheter advancement between 5 and 10 cm was likely due to contact with the inferior wall of the arch of aorta, requiring manipulation. If no resistance was felt by 20 cm, then it was likely to have advanced down the descending aorta requiring repositioning if possible. When the catheter advanced correctly down the ascending aorta, the catheter often 'bounced' at about 10-15 cm on the leaflets of the aortic valve and could then be pulled back slightly. A guide wire occasionally was used to help stiffen the catheter. The balloon was then inflated above the ostia in the ascending aorta to prevent the flow of contrast up the ascending aorta. The balloon was inflated with dilute water-soluble radiographic contrast (1 in 50 dilution of Urografin®) to help ascertain the position of the balloon in the aorta on initial scans.

PMCT was undertaken the same evening as recruitment and cannulation, using a Toshiba Aquilion 64 slice scanner (120 kVp, 300 mA and 64 x 0·5 mm slice thickness, pitch 0·83, matrix 512 x 512). Pre-contrast scans were performed in three overlapping blocks of "head & neck", "chest, abdomen and pelvis" and "pelvis and legs". The head and neck were scanned with both straight and angled tube to offset dental metal artefact to different levels. Slices were reconstructed to 1mm (head and chest) or 2mm (abdomen and pelvis) slices using both soft tissue and bone algorithm. Boost (metal artefact reduction) was on for all scans. Auto mA was not used. A large field of view was used for body, as arm and limb positioning could be difficult. Contrast runs through the heart were reconstructed at 0·5mm with a reduced field of view. Five separate sequences were performed using air (negative contrast) for the first three sequences, followed by two sequences of Urografin® 150 mg/ml (Bayer Healthcare, positive contrast) diluted 1:10 as previously described. The first 150 cases involved manual injection via a standard 60 ml bladder syringe using gentle 'constant' hand pressure, before changing to a Medrad Stellant dual head pump injector system (Medrad UK Ltd, UK). [4]

Air injection: Pump injector 300mls air at 6mls/sec, 43 second delay from start of injection to start of imaging. For hand injection 5 x 60 mls bladder syringe over 2 – 3 minutes. Positive contrast medium (Urografin® 150 mg/ml, Bayer Healthcare) diluted 1:10 at 150mls at 3mls per second, 43-second delay from start of injection to start of imaging. For hand injection: 120 ml of positive contrast in 2 injections in approximately 40 seconds (20 seconds per syringe).

The anonymized DICOM image set was analysed either on an Agfa Impax 6·5 workstation © Agfa HealthCare Corp. USA, or an Apple Mac Pro workstation using OsiriX v4·0 64-bit software (Pixmeo, Switzerland), both with Multi-Plane Reconstruction (MPR), curved MPR and volume rendering capability.

Supplementary figure S1: Study profile

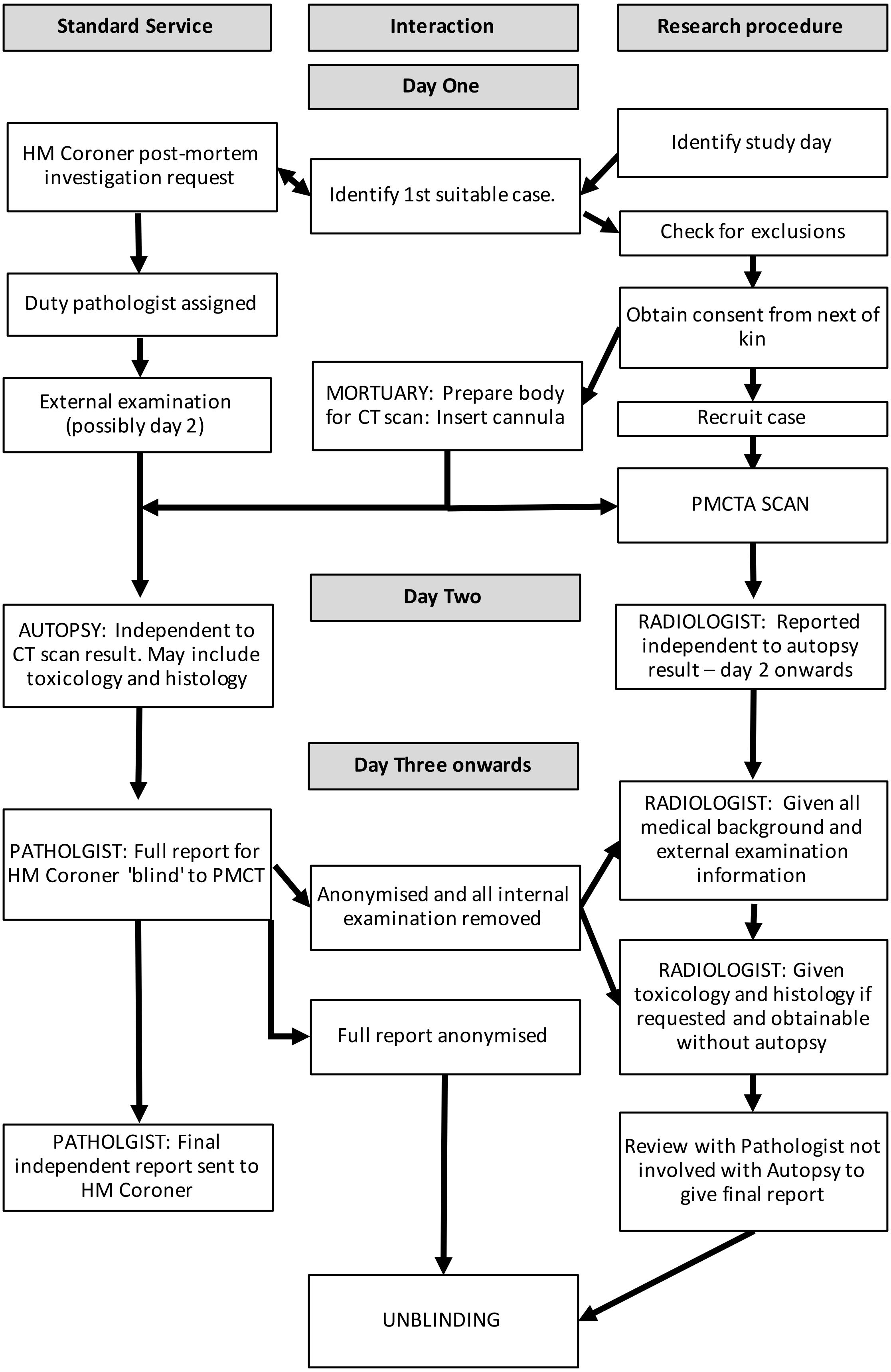

Supplementary Text: The gold standard

The "gold standard" result was taken from the autopsy cause of death (CoD) and findings unless modified by one of four factors:

1: PMCTA showed a clear and incontrovertible finding, such as fracture or major haemorrhage, where PMCT can be considered specific.

2: A significant finding on PMCT is confirmed by autopsy, but increases the significance of the autopsy finding. This is therefore added to the CoD. For example in one case, pneumoperitoneum was seen on PMCT without evidence of decomposition (lack of portal vein gas). On autopsy the pneumoperitoneum was not identified, but autopsy did show an area of abnormal bowel wall with gas within it. Perforated viscous was therefore added to the CoD, although both tests agreed that significant cardiovascular disease was present and the major factor.

3: Specific pre-mortem investigations and findings, not appreciated during the autopsy investigation, contradict diagnosis. For example cardiomegaly changed hypertrophic cardiomyopathy (HOCM) in the presence of pre-mortem diagnosis of HOCM, based on pre-mortem cardiac imaging after the death of a 1st degree relative from HOCM.

4: CoD constructed incorrectly, based on the autopsy findings. These were generally changes for consistency, did not result in discrepancies, and were therefore considered trivial.

Supplementary Text: Defining discrepancies between PMCTA, autopsy and the gold standard

Differences in autopsy and PMCT reports were categorised into major, minor (Supplementary Figure S2), no discrepancy, or were recorded separately. Major discrepancies included missing significant trauma, ascribing CoD to the wrong organ or a very different mechanism, or missing any significant potentially fatal finding, even if not the CoD. Minor discrepancies included CoD related to wrong organ system; but with no discrepancy in findings, the same organ system; with linked but different cause, trauma not directly relevant to CoD, and failure to find a second condition that may have contributed to death. Other anomalies, such as reversed order of CoD, two diagnoses instead of one; if there were no discrepancy in findings, and failure to mention common incidental pathologies, were not considered discrepant. The diagnoses of ischaemic heart disease, coronary artery disease, myocardial infarction, and coronary thrombosis were considered equivalent. Fractures sustained as part of resuscitation, left ventricular hypertrophy as a secondary finding, incidental pleural fluid or mild ascites not reported on autopsy report, and old cerebral infarcts not relevant to cause of death were recorded separately and not categorised as discrepancies (Supplementary Table S1).

Supplementary Figure S2: Example of one major and one minor discrepancy.

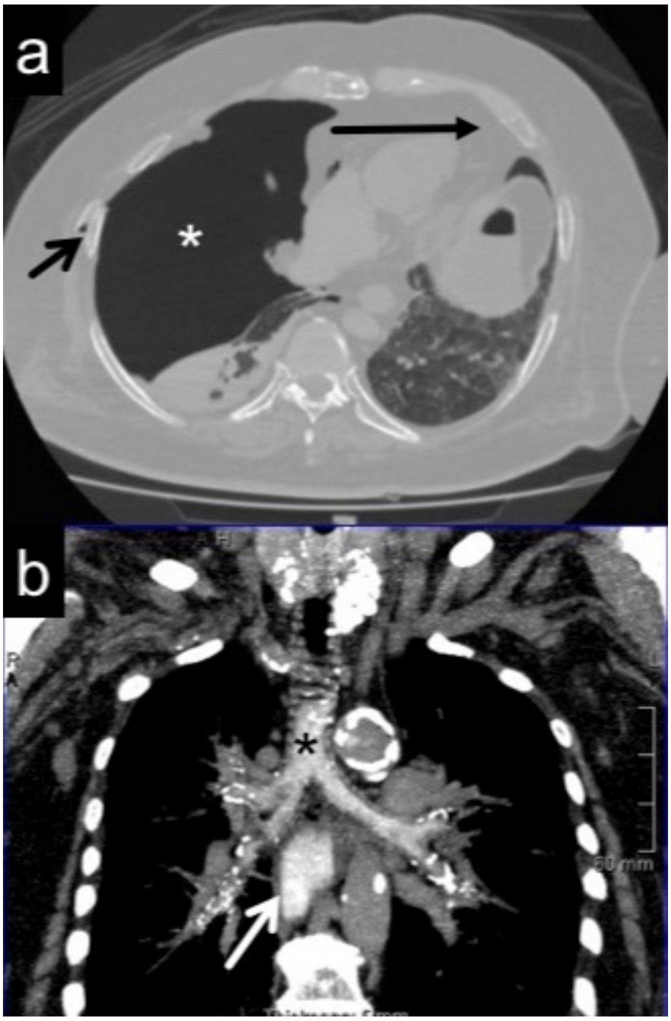

Legend to Figure S2: Figure S2a shows an axial chest PMCT image with rib fracture (open arrow) and tension pneumothorax (* and closed arrow) in a 67-year male found dead at home after not being seen for several days. There was no resuscitation attempt. Toxicology was consistent with significant diabetic ketoacidosis (DKA), but autopsy report does not mention the traumatic findings and gives CoD as DKA alone, which is considered a major discrepancy. Figure S2b is an 82-year female who died of myocardial insufficiency after an operation to fix a fractured femur agreed by autopsy and PMCT. However, PMCT clearly showed the patient had aspirated gastric contents into the major airways (*) from the stomach and oesophagus (arrow), demonstrated because the patient had radio opaque gastric contents, probably from ingesting antacid medication (which can be radio opaque). This corresponded with the clinical notes that recorded possible aspiration prior to her final deterioration. This was considered a minor discrepancy as aspiration was not considered the most important reason for her death.

Supplementary Table S1: Defining degree of discrepancy in Cause of Death and findings. For the purpose of analysis certain other common themes were recorded independently.

	Discrepancy	Example
**Major**	Significant Trauma relevant to CoD	
	CoD related to wrong organ system or very different mechanism	PE Vs Heart disease<br>Lung cancer Vs pneumonia
	Missing any significant potentially fatal finding, even if not the CoD	Significant haemorrhage or pneumothorax
**Minor**	CoD related to wrong organ system on balance of probabilities BUT with no discrepancy in findings	Both tests report significant heart and lung disease, but ascribe CoD differently
	Same organ system with linked but different cause	IHD Vs hypertensive heart disease<br>Bronchopneumonia Vs COPD.
	Significant trauma not directly relevant to CoD	Fractured extremity
	Failure to find a second condition that may have contributed to death	PE and IHD Vs PE, where only one test documents IHD
**Not discrepant**	Order of CoD reversed	1a: IHD 2: COPD Vs 1a: COPD 2: IHD
	Minor differences in detail	Bronchopneumonia Vs lobar pneumonia
	Two diagnoses instead of one if there are no discrepancy in findings	PE and IHD Vs PE, when both tests report IHD
	Failure to mention a chronic condition in part 2 that is clear in the medical record	Known Diabetes mellitus or hypertension
	Failure to mention common incidental pathology	Age related changes, simple renal cysts, enlarged prostate gland, and uncomplicated diverticulosis.
**Recorded independently**	Critical IHD or coronary artery disease Vs MI or coronary thrombosis (**footnote 1**)	
	Left ventricular hypertrophy as a secondary finding (**footnote 2**)	
	Fractures sustained as part of resuscitation or Incidental pleural fluid or mild ascites not reported on autopsy report (**footnote 3**)	
	Old Cerebral infarcts not relevant to CoD (**footnote 4**)	

IHD – Ischemic Heart Disease, PE – Pulmonary thromboembolism, COPD – Chronic Obstructive Pulmonary Disease, MI – Myocardial infarction, CoD – Cause of Death

Footnote 1 to Table S1: Death due to "ischaemic heart disease" was not considered discrepant to coronary artery thrombosis or myocardial infarction. Although PMCTA can detect occlusions or critical stenosis of the coronary arteries it is generally insensitive to acute myocardial infarction, although the diagnosis can sometimes be made (see Figure 1, main text).

Footnote 2 to Table S1: Disagreement about left ventricular hypertrophy (LVH) as a finding when not the primary CoD. PMCTA with air contrast defines the left ventricular wall well and may contradict the autopsy findings (Supplementary Figure S3**).**

Footnote 3 to Table S1: Autopsy reports often failed to mention anterior rib fractures due to CPR and pleural effusions or ascites clearly visible on PMCT. These were only counted in the discrepancy analysis if important to the CoD.

Footnote 4 to Table S1: Old cerebral infarcts were frequently reported on imaging, but not always recorded in the autopsy report. Cerebral infarcts were only recorded as a major discrepancy if they were directly relevant to CoD (Supplementary Figure S4).

Supplementary Figure S3: Left ventricular hypertrophy (LVH) (two cases).

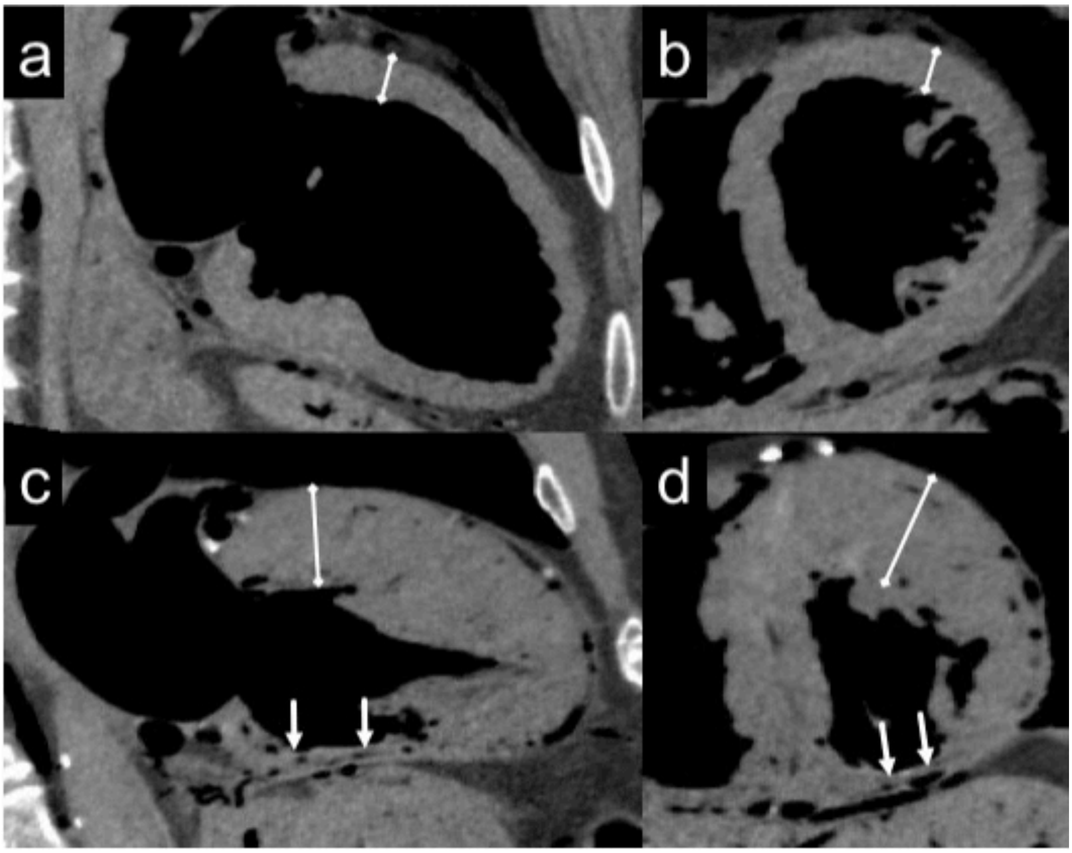

Legend to Figure S3: Short and long axis MPR reconstructions of the left ventricle after air contrast runs providing ventriculograms. Fig. S3a&b show a 59-year male with a history of hypertension, where autopsy reports LVH despite normal myocardial thickness (double ended arrows) on imaging, making significant LVH improbable. In the second case (fig. S3c&d) a 76-year male with a history of hypertension and previous MI, autopsy and PMCTA agreed on the diagnosis of significant LVH secondary to hypertension. There was also agreement on a region of myocardial thinning (arrows) secondary to old myocardial infarction.

Supplementary Figure S4: Cerebral infarction (two cases).

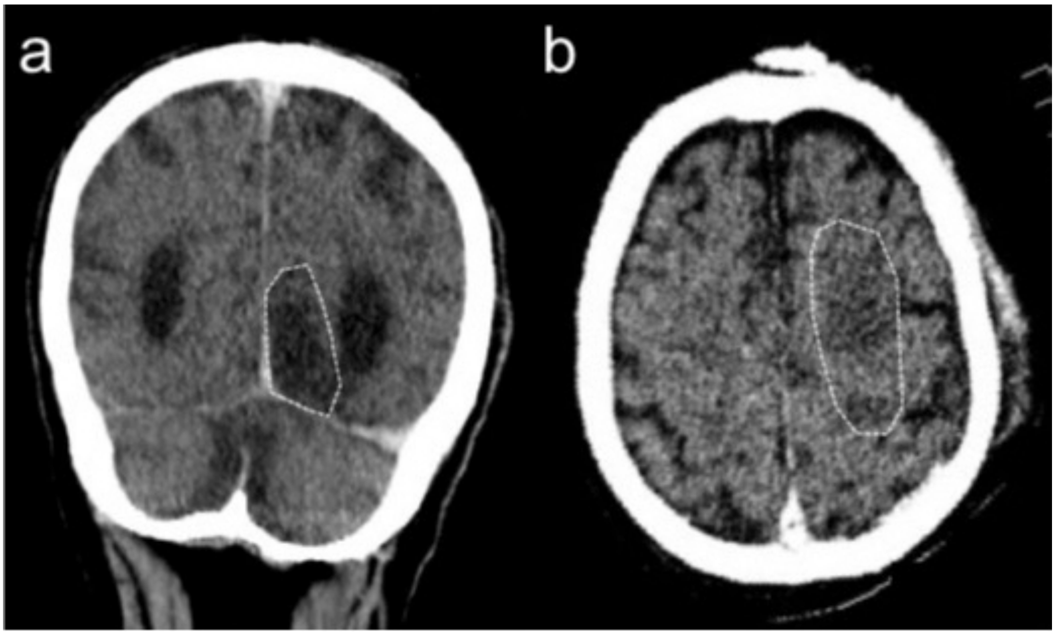

Legend to Figure S4: Fig. S4a shows a coronal image of the brain of an 83-year male who died of acute ruptured abdominal aortic aneurysm. Autopsy report did not mention an old occipital infarct (dashed line) clearly demonstrated on PMCT. This was considered a minor discrepancy, as it was not related to CoD. Conversely, figure S4b shows axial brain image of an 86-year female with low density in the left cerebral white matter (dashed line) corresponding to an acute cerebral infarct on autopsy. Although visible in retrospect on PMCT, this was not reported and CoD was attributed to significant coronary artery disease, which was also present on autopsy and given in part 2. This counted as a major discrepancy although "on another day" the radiologist may have given a co-diagnosis of coronary and cerebrovascular disease and been in agreement with autopsy.

Supplementary Results: Toxicology, biochemistry, microbiology and histology testing

Toxicology and biochemistry

The PMCTA reporting team requested toxicology & biochemistry in 38/210 cases (18·1%) cases: thirty-two (15·4%) at triage; performed in 29/32 (90·6%), and seven (3·3%) after reviewing the PMCTA scan; performed in 2/7 (28·6%). Therefore, in 31/210 (14·8%) cases, including sixteen due to a definite history of suspected high ethanol and/or drug intake, data were provided to the PMCTA reporters. In 21/31 (68%) cases where performed, toxicology and/or biochemistry were critical to diagnosing the CoD. However, in the seven cases where toxicology was requested after reviewing the PMCTA scan, it was only done in two cases and was unhelpful. Supplementary table S3 gives the breakdown of toxicology/biochemistry cases.

Supplementary table S2: Breakdown of reasons for toxicology / biochemistry performed.

Reason	Why Performed	Why Requested	CoD given	Triage to autopsy	PMCT discrepancy		Autopsy discrepancy	
					Minor	Major	Minor	Major
Alcohol or Drugs	16	16	15	1	1	1	2	
Trauma	3	2	3	0				
Possible suspicious circumstances	3	3	3	0				
Do not know CoD	5	3	2	3				
IDDM	6	6	6	0	1		1	2
Epilepsy	2	2	2	1	1		1	
Possible allergy issues	1	0	1	0				
Don't know why	4	0	4	0				
Total performed*	40	32	36	5	3	1	4	2
Per cent (%)			12·5	7·5	2·5	10·0	5·0	12·5
Not performed *	170	7	157	31	18	11	9	7
Per cent (%)			18·2	10·6	6·5	5·3	4·1	18·2
All cases	210	39	193	36	21	12	13	9

CoD: Cause of Death, IDDM: Insulin Dependent Diabetes Mellitus

* There was no statistical difference between the rates of given "cause of death", "triage to autopsy" decision or discrepancy between the toxicology and non-toxicology groups.

Histology and Microbiology

The PMCTA reporting team requested histology that could have been obtained by CT guided biopsy, in 15 (7·1%) cases: seven at triage and eight after PMCTA, for heart in six cases, lung in six cases, tuberculosis in one case, pancreas in one case and microbiology screen for sepsis source in one case. In ten of these cases, histology was performed at autopsy and was available. In five of these cases, histology confirmed expected pathology of asbestosis or cancer. In two cases, it directly affected the autopsy cause of death (meningitis and pulmonary infection). In three cases it was not diagnostic. In a further 6 cases not specifically requested, but where histology was done, the PMCT reporters had decided to "triage to autopsy", so it is not known whether they may have requested specific CT guided biopsy. Histology was actually performed in 38 cases, but was not seen by the PMCTA reporting team if not specifically requested.

Supplementary table S3: Breakdown of reasons for histology / microbiology performed.

Reason		Done	Requested by PMCTA team	NR-TTA	COD given	Triage to autopsy	PMCT discrepancy		Autopsy discrepancy	
							Minor	Major	Minor	Major
Diagnosis not known										
	Prob. Cardiac	8	3	2	3	5	3	0	0	0
	Prob. lung	2	0	1	1	1	1	0	2	0
Clarify Diagnosis										
	Heart	2	0	0	2	0	0	1	0	0
	Cancer	8	3	1	8	2	2	0	1	0
	Lung	3	0		3	0	0	0	0	0
Industrial lung disease or Mesothelioma		4	3	0	4	0	0	0	0	0
	Sepsis / Infection	2	2	0	2	1	1	0	0	1
	Brain	2		2	2	2	0	0	0	0
No specific reason identified										
	Multi organ failure	2	0	0	2	0	0	0	0	0
	Choking	1	0	0	1	0	0	0	0	0
	Prob. Associated with Toxicology	4	0	0	4	0	0	0	0	0
Total Performed		38	11	6	32	11	3	1	2	1
Per cent (%)					84·2^{+}	28·9^{++}	7·9	2·6	5·3	2·6
Not Performed		172	4*		161	25	18	11	11	8
Per cent (%)					93·6^{+}	14·5^{++}	10·5	6·4	6·4	4·7
Total cases		210			193	36	21	12	13	9

CoD: Cause of death, NR-TTA: Histology / microbiology not requested specifically, but case "triaged to autopsy" so it may have been requested if autopsy were not available.

*The four cases requested, but not performed, were to clarify pancreatitis in one case, to clarify cardiac problem in two cases, and in the other was due to trauma leading to possible hypothermia in a probable cardiac death, in which the trauma was not recognised at autopsy. All cases were given a CoD with no major discrepancy, but 3/4 of these cases were "triaged to autopsy".

The per cent where a cause of death was given was lower (+ p=0·092) and the per cent "triage to autopsy" was higher (++ p=0·054) in the histology-performed group. Although these values are not statistically significant, the difference would be expected, relating to cases where autopsy and histology were required to make a diagnosis.

There was no statistical difference between the rates of discrepancy.

Potential impact of requirement Toxicology, biochemistry, microbiology and histology testing on ability to use PMCTA as an alternative to autopsy

There were two cases where the autopsy pathologist was informed of CT scan findings, one case where TB was first suspected during the CT scan vascular cannulation [5] and one case where the pathologist requested information regarding chest trauma and pneumothorax. In four cases, the trainee pathologist who supervised the cannulation, contrast injection, and scan was also present at the autopsy. They were not trained in image interpretation and did not have access to the radiologist's opinion, but could have influenced the autopsy result.

Regardless of whether performed or not, forty-two cases had either toxicology/biochemistry requested based on the history at triage, or histology/microbiology requested, based on the history and/or PMCTA scan, and if accessible by CT guided biopsy (32 toxicology, 15 histology, and 5 cases of both). A CoD was given in 40/210 (19·0%) of these cases. PMCTA services may not wish to include cases where further tests are predicted to be required, unless rapid testing is available, because theoretically the delayed result may show the need for autopsy, leading to delayed release of the body to the next-of-kin. In this study a CoD was given in 19% cases after predictable further tests, and therefore these 19% of cases could be deemed unsuitable for a PMCTA service. The impact of these tests could only be assessed if they were performed. Suppl. table S5 shows that if toxicology / biochemistry and histology / microbiology were requested for specific reasons (as opposed to simply not finding a CoD found on PMCTA) and were performed, the combination of PMCTA and the test result was normally sufficient to provide an acceptable CoD on the balance of probabilities.

Supplementary table S4: Breakdown of discrepancy rate where histology / microbiology or toxicology / biochemistry were requested and performed for specific reasons.

	Number requested and performed	CoD Given	PMCT discrepancy		Autopsy discrepancy	
			Minor	Major	Minor	Major
Toxicology	29	28	3	1	4	2
Histology	9	9	2	0	1	1
Total (both)	38	37**	5	1	5	3
Per cent (%)		97.4	13·2	2·6	13·2	7·9
Overall	210	193	21	12	13	9
Per cent (%)*		91·9	10·9	6·2	6·7	4·7

CoD: Cause of death

* Overall per cent (%) calculated based on cases where a CoD was given (as per table 3 in the main manuscript).

There is no statistically significant difference between the discrepancy rates for the Toxicology / Histology group compared with overall discrepancy rates.

** In one case no CoD was given. Toxicology and biochemistry were unhelpful. Both tests showed equivalent coronary vascular disease, which autopsy gave as the CoD on balance of probabilities.

Triage to autopsy decisions and failure to give a cause of death after PMCTA

After review of PMCTA the reporting team gave a CoD in 193/210 cases (92%). Of these 193 cases, the review team signalled lower confidence in 19 cases where, despite giving a CoD, they would still have "triaged to autopsy".

Supplementary Table S5: Reasons for not giving a cause of death or deciding to "triage to autopsy" based on low confidence.

	Decision after PMCTA	
	**No Cause of death**	**"Triage to Autopsy"**
**TOTAL**	17	36
**Failed angiogram and considered cardiac**	5	11
**Histology required but not available to PMCT**	0	4
**Complex Case**	4	6
**Possible traumatic cause of SAH**	0	1
**Low Confidence**	0	1
**Pulmonary thromboembolism considered, but unsure**	3	7
**No abnormality sufficient to cause death seen**	5	5
**To locate source of sepsis**	0	1

Note: The "No cause of death" given group is a subset of the "triage to autopsy group".

In the 17 cases where a CoD was not given by PMCTA, at least seven were felt to be diagnoses of exclusion on autopsy. For example, PMCTA and autopsy may have agreed on the findings, such as left ventricular hypertrophy with a medical history of hypertension, but whilst PMCTA failed to reach a conclusion, autopsy gave a diagnosis based on the balance of probabilities, through necessity.

Supplementary Table S6: Sensitivity and specificity results for findings and cause of death for Autopsy and "PMCT approach" (in % with 95% confidence intervals below).

Diagnosis	Pulmonary Thromboembolism	Trauma	Cardiac	Respiratory
**Prevalence**	6.2	7.6	53·8	16·2
**Sensitivity PMCT**	54*	100**	97	91
	(25 – 81)	(79 – 100)	(92 – 99)	(76 – 98)
**Sensitivity Autopsy**	100*	56**	99	92
	(75 – 100)	(30 – 80)	(95 – 100)	(77 – 98)
**Specificity PMCT**	98	100	100	98
	(96 – 100)	(98 – 100)	(96 – 100)	(94 – 99)
**Specificity Autopsy**	100	100	100	100
	(98 – 100)	(98 – 100)	(96 – 100)	(98 – 100)

Overall sensitivity and specificity for pulmonary thromboembolism, trauma, respiratory disease, and cardiac disease are shown in table A4. This compares autopsy findings and the "PMCT approach" with the "gold standard"> the PMCT approach uses the PMCTA findings alone in the 193/210 cases where a CoD is given and both the PMCT and autopsy findings in the 17/210 cases where PMCTA does not give a CoD.

Difference significant (McNemar's test) * p=0·004, ** p=0·016.

Supplementary text: Overall population statistics for cause of death.

Supplementary Table S8 shows a summary of causes of death obtained using different approaches based on: autopsy alone, PMCTA + autopsy only when no CoD given, and the gold standard. These data show no statistically significant difference in reported pathology rates between the different diagnostic pathways. As expected from table S7, PMCTA reports less pulmonary thromboembolism (PE), and autopsy less trauma. The total diagnoses exceed numbers of cases, as multiple pathologies may exist. Although PMCTA diagnoses fewer 'PE's, the figures are balanced by false positives, and the numbers of PE where autopsy results are used when PMCTA gives no CoD. PMCT recorded 4 fewer cancer diagnoses, but in all cases the abnormality was demonstrated, one case was missed by the radiologist, one case ignored as irrelevant, one case was called pneumonia and in one case called either cancer or ulcer disease in an esophageal perforation. In most discrepancy cases the missed trauma was considered a contributory rather than primary factor to the CoD.

Supplementary Table S7: Population statistics for cause of death (CoD). These show no significant difference in reported pathology rates between the different diagnostic pathways although autopsy diagnoses less trauma and PMCT less PE.

**Cause of death**	**Gold Standard**	**Autopsy**	**PMCT or autopsy if CoD not given.**
Cardiac	147	152	147
Great vessel Hemorrhage	14	14	15
Respiratory	30	29	28
PE	14	13	10
Trauma	16	10	16
Brain	11	8	9
Abdomen and GI tract	19	18	17
Diabetic Ketoacidosis	4	4	4
Poisoning	4	4	4
Alcohol Related	12	10	14
Advanced Cancer	19	19	15
Sepsis (non lung)	2	2	2
Post operative complications	7	7	7
Inherited	1	1	1
Smoke inhalation	2	2	2
**Total**	302	293	291

References

1. Grabherr S, Doenz F, Steger B, et al. Multi-phase post-mortem CT angiography: development of a standardized protocol. *Int J Legal Med* 2011; **125**(6): 791-802.

2. Morgan B, Sakamoto N, Shiotani S, Grabherr S. Postmortem computed tomography (PMCT) scanning with angiography (PMCTA): a description of three distinct methods. In: Rutty GN, editor. Essential of Autopsy Practice. Advances, updates and Emerging Technologies. Springer, London,; 2014. p.1-22.

3. Saunders SL, Morgan B, Raj V, Robinson CE, Rutty GN. Targeted post-mortem computed tomography cardiac angiography: proof of concept. *Int J Legal Med* 2011; **125**(4): 609-16.

4. Robinson C, Barber J, Amoroso J, Morgan B, Rutty G. Pump injector system applied to targeted post-mortem coronary artery angiography. *Int J Legal Med* 2013; **127**(3): 661-6.

5. Clarke M, McGregor A, Robinson C, Amoroso J, Morgan B, Rutty G. Identifying the correct cause of death: The role of post-mortem computed tomography in sudden unexplained death. . *J Forensic Radiol Imaging* 2014; **2**: 210-2.
